# What can cohort studies in the dog tell us?

**DOI:** 10.1186/2052-6687-1-5

**Published:** 2014-05-15

**Authors:** Carys A Pugh, Barend M de C Bronsvoort, Ian G Handel, Kim M Summers, Dylan N Clements

**Affiliations:** The Roslin Institute and The Royal (Dick) School of Veterinary Studies, The University of Edinburgh, Easter Bush, Midlothian, EH25 9RG UK

**Keywords:** Canine, Cohort, Longitudinal, Epidemiology

## Abstract

This paper addresses the use of cohort studies in canine medicine to date and highlights the benefits of wider use of such studies in the future. Uniquely amongst observational studies, cohort studies offer the investigator an opportunity to assess the temporal relationship between hypothesised risk factors and diseases. In human medicine cohort studies were initially used to investigate specific exposures but there has been a movement in recent years to more broadly assess the impact of complex lifestyles on morbidity and mortality. Such studies do not focus on narrow prior hypotheses but rather generate new theories about the impact of environmental and genetic risk factors on disease. Unfortunately cohort studies are expensive both in terms of initial investment and on-going costs. There is inevitably a delay between set up and the reporting of meaningful results. Expense and time constraints are likely why this study design has been used sparingly in the field of canine health studies. Despite their rather limited numbers, canine cohort studies have made a valuable contribution to the understanding of dog health, in areas such as the dynamics of infectious disease. Individual exposures such as neutering and dietary restriction have also been directly investigated. More recently, following the trend in human health, large cohort studies have been set up to assess the wider impact of dog lifestyle on their health. Such studies have the potential to develop and test hypotheses and stimulate new theories regarding the maintenance of life-long health in canine populations.

## Lay summary

Cohort studies involve the repeated collection of information through time. Investigators are able to assess whether exposure to a particular risk factor (these could be environmental, diet, lifestyle, genetic etc) is associated with, and followed by, clinical outcomes in individuals. This paper highlights the benefits of wider use of such studies in the future. Thus researchers collect information (e.g. clinical data and records), rather than DNA samples to use in genetic studies. Information is longitudinally collected at regular time intervals, and allows investigators to assess whether exposure to particular risk factors (these could be environmental, diet, lifestyle, genetic, etc) are associated with, and followed by, clinical outcomes in individuals. This paper highlights the benefits of wider use of such studies in the future.

Uniquely amongst observational studies, cohort studies offer researchers an opportunity to assess the time relationship between suggested risk factors and diseases. In human medicine, cohort studies were originally used to investigate specific exposures but there has been a recent move to more broadly assessing the impact of complex lifestyles on life and death. Such studies can generate new theories about the impact of environmental and genetic risk factors on disease. However, cohort studies are expensive and there is, inevitably, a delay between setting them up and the final reporting of meaningful results. Expense and time constraints are why this type of study has been used sparingly in canine health studies. Despite limited numbers, canine cohort studies have made a valuable contribution to the understanding of dog health, in areas such as infectious disease. Individual exposures such as neutering and dietary restriction have also been investigated. More recently, large cohort studies have been set up to assess the impact of dog lifestyle on their health. Such studies have the potential to develop and test hypotheses (ideas) and stimulate new theories regarding the maintenance of life-long health in canine populations.

## Introduction

Understanding the factors relating to disease in a population is important for anticipating and dealing with health care needs. The health of populations can be studied in a number of ways. Beyond descriptive approaches, analytical studies can be split into experimental and observational investigations. Dohoo *et al.*[1] distinguished observational studies from experimental studies, where investigators control the allocation of subjects to study groups, by suggesting that in observational studies, investigators “try not to influence the natural course of events for the study subjects”.

Epidemiologists traditional divide observational studies into case–control, cross-sectional or cohort study designs [1, 2]. The advantages and disadvantages of each of these study types, particularly with regard to susceptibility to bias, are fully described in Table 1. In brief however, case–control studies are particularly useful for rare diseases but lack an ability to clarify temporal relationships between events and exposures. Cross-sectional studies can be performed at a single time point and allow investigators to seek associations between potential risk factors and outcomes, but again do not allow the assessment of temporal dependencies. Cohort studies, where individuals are tracked through time, solve this problem as investigators can assess whether risk factor exposures are *followed* by outcomes in individuals. This element of time dependency is crucial to infer causation between risk factors and disease, and to understand transmission dynamics of infectious diseases. Further, cohort studies lend themselves to analysis of the effect of long-term exposure to a risk factor or treatment and, with targeted recruitment, are ideally suited to examine the effect of rare risk factors. Unfortunately cohort studies necessarily involve a large investment of time and finances, both to set up and maintain, and have therefore been used sparingly in the field of canine disease. In this review we will discuss studies found in broader medical literature before describing the types of canine cohort studies reported to date. The techniques found in human medicine may be applied to canine epidemiology with immense potential for health advances.

**Table 1:** **The advantages and disadvantages of different observational study types**

Study type	Potential goals	Advantages	Disadvantages
Cross-sectional	- Population prevalence of exposure and/or outcome	- Relatively simple	- Poor for rarer exposures and outcomes
	- Associations between exposures and outcomes	- Relatively cheap	- No causality may be inferred as exposures and outcomes are measured contemporaneously
		- Relatively quick	- Highly susceptible to information bias
		- Good for common conditions and exposures	
		- May assess multiple exposures and outcomes	
		- Good for initial assessment of an exposure or outcome	
Case–control	- Associations between exposures and outcome	- Relatively cheap	- Choice of controls notoriously difficult
	- Strength of association in the form of odds ratio between exposure(s) in controls and exposure(s) in cases	- Relatively quick	- May only examine one outcome
		- May assess long latent periods	- Odds ratio not an intuitive measure
		- Good for rarer outcomes	- Highly susceptible to selection and information bias and population stratification
Cohort	- Incidence rates	- Good for rare exposures	- Not simple
	- Temporal associations between exposures and outcomes	- May examine multiple exposures and outcomes	- Not cheap
		- May assess long latent periods	- Not quick (unless retrospective)
		- May assess temporal relationship between exposure and outcome inferring causality	- Highly susceptible to retention bias
			- Susceptible to sampling bias

## Review

### The benefits of cohort studies: comparative examples

One of the most widely renowned cohort studies in human medicine is the Framingham Heart Study. Researchers recruited a group of over 5,000 women and men aged between 30 and 62 years old living in Framingham, Massachusetts in 1948. The cohort were evaluated every two years regarding their medical status and lifestyles, including physical examinations and collection of biological samples for laboratory testing. The study identified many of the major cardiovascular disease risk factors which we take for granted today, such as high blood pressure, high blood cholesterol, smoking, obesity and diabetes [3]. The analysis of the Framingham cohort has resulted in over 2,000 peer reviewed publications, and aptly demonstrates how the detailed, repeated evaluation of modestly sized cohort groups can result in the identification of risk factors for disease which have global significance.

Another early cohort study of human health was undertaken in the UK in 1951. The aim was to address concerns about an observed association between smoking and lung cancer. To examine the question of causality, the study was designed to determine whether it was possible to predict someone’s risk of developing lung cancer from their smoking habits earlier in life [4]. Over 40,000 doctors were recruited, which was over two thirds of the doctors on the British Medical Register at the time. The study went on to investigate the impact of smoking on diseases beyond lung cancer, including vascular disease and other neoplasias [5]. Ultimately the cohort was so valuable that the members were followed for their lifetime and the last questionnaire was sent out some 50 years later.

Two more recent studies which have illustrated the power of large scale cohort studies are the Avon Longitudinal Study of Parents and Children (ALSPAC) [6] and the Italian NINFEA cohort [7]. Both are birth cohorts, initially designed without specific hypotheses in mind. Instead they set out to collect information on a variety of exposures to broadly investigate pregnancy and the early life of children. In the case of ALSPAC, investigations went back even earlier, with assessment of antenatal risks, such as the impact of maternal drinking prior to conception and in early pregnancy on birth weight [8].

The ALSPAC study team faced great difficulty obtaining funding in the initial years of the project [9]. As time passed and significant risk factors started to be found and reported, it became more widely recognised that the cohort was an incredible resource that should be maintained in the long-term. This open-ended investigative approach resulted in the identification of a range of phenotypes and influencing factors that could not have been predicted by the investigators at the start of the study. The costs of recruiting the cohort would have been wasted if contact with members were lost before these discoveries could be made.

Analyses of the ALSPAC cohort did not stop with exploration of early-life influences. As the costs of collecting, archiving and analysing DNA reduced it became possible to add genetic data to the wealth of phenotypic data and explore the interaction of genotype with other variables. Over more than 20 years the ALSPAC team moved from having a relationship with pregnant mothers to having a relationship with the children from those pregnancies. These children have grown to start their own families and the next generation are also being recruited into the study. A wealth of discoveries guiding national public health policy have been made during the study. These include understanding the influence of sleeping position on the risk of cot death [10, 11] and the benefits of eating oily fish on children’s mental development [12], both of which have directly led to the development of guidelines for best practice.

Between 1996 and 2001, the Million Women Study recruited women over 50 in the UK [13]. Recruitment through breast cancer screening centres built-in a reliable method of ascertaining the primary outcome of interest – the incidence of breast cancer. Environmental influences were captured in a lifestyle questionnaire that was completed at recruitment and periodically thereafter. Information regarding other disease events such as incidence of fractures was also collected via the follow-up questionnaires [14].

The main finding from the Million Women Study regarding the impact of Hormone Replacement Therapy (HRT) on the incidence of breast cancer [15] remains controversial. An increased incidence of breast cancer was found in the women taking HRT but it has subsequently been argued that these women were more likely to be tested for breast cancer, resulting in increased diagnoses. The women involved were not randomly assigned to receive HRT so the potential for confounding cannot be ignored. Nevertheless, the study built on results from earlier cross-sectional studies and it had enormous power to detect associations. As the women were followed with time, causal inference is possible. At the very least the results of the many publications about the cohort will influence the direction of future randomised controlled trials to try and definitively determine causal relationships.

The value of cohorts has been recognised and data collected previously are increasingly the foundation for further analysis. For example, a team from Edinburgh University took advantage of historic data collection to develop a cohort of people with results that span over 80 years. In Scotland 95% of children born in 1921 and 1936 were given an intelligence test at the age of 11. The team recruited a subset of the survivors from these tested cohorts some 60–70 years later to investigate their cognitive function [16] and the environmental and genetic influences upon them. Their continuing assessment of cognitive function has led to the discovery of an association between carrying the *APOE* E4 allele (also associated with Alzheimer’s disease) and non-pathological cognitive decline [17]. The cohort is a unique resource for the investigation of the effects of aging on cognition and it continues as participants enter their tenth decade.

While the benefits of cohort studies are well understood (Table 1), the extended time to finding results and relatively high costs are undeniable. In part to address these costs, the US Department of Defense started to move cohort studies into the internet age when they set up the Millennium Cohort Study of US military personnel [18]. Current and ex-military personnel were recruited and offered the chance to answer the questionnaire by post or online. The financial savings associated with participants replying online were such that they offered a $5 incentive and still estimated their savings per online response at $50 compared to those responding by post [19]. As internet access has increased, epidemiological studies have gradually made greater use of the technology. The NINFEA cohort is based entirely online [20]. Whilst the costs of setting up and maintaining functional and appealing web portals are not insignificant, studies are now possible that would not have been feasible if based on face-to-face, telephone or postal questionnaires. Building on this experience of human studies, canine cohort studies that would have been inconceivable are now financially viable and the potential to exploit this avenue of research is immense.

## Canine cohort studies

Despite the extensive number of findings uncovered by human cohort studies, the design has not been widely used in canine research in the past. As discussed, the cost and time burdens can be prohibitively high. A number of canine cohort studies have been reported and in each case attempts have been made to overcome the associated financial burden. The different strategies used are discussed below and their merits summarised in Table 2.

**Table 2:** **The advantages and disadvantages of different cohort study types**

Study type	Data source(s)	Advantages	Disadvantages
Retrospective	Pre-existing insurance databases	- Relatively cheap	- Non-standardised diagnostic criteria
		- Relatively quick	- Poor generalisability in countries with high uninsured population
		- May assess multiple clinical exposures and outcomes	- No requirement for insurance data to be made available
		- May assess long latent periods	
		- Recruitment and retention simple	
	Pre-existing databases from secondary veterinary hospitals	- Relatively cheap	- Non-standardised diagnostic criteria
		- Relatively quick	- Non-standardised recording systems
		- May assess multiple clinical exposures and outcomes	- No knowledge of wider environmental exposures
		- Potential to use ancillary resources	- Potential for referral and geographical bias
		- May assess long latent periods	
		- Good for examining serious illnesses	
		- Recruitment and retention simple	
	Pre-existing databases from primary veterinary clinics	- Relatively cheap	- Non-standardised diagnostic criteria
		- Relatively quick	- Non-standardised recording systems
		- May assess multiple clinical exposures and outcomes	- No knowledge of wider environmental exposures
		- Recruitment simple	- Potential for retention bias as owners move practices
Prospective: Time Limited	According to study protocol: May include investigators, veterinarians, breeders and owners	- Costs and time limited according to length of the study	- Necessarily time limited so unable to assess long-term exposures and long latent periods
		- May assess multiple exposures and outcomes including wider environmental exposures	- Recruitment not simple
		- Good for the study of infectious diseases	
		- Diagnostic criteria set according to study protocol	
		- Retention bias is minimised	
Prospective: Single issue	According to study protocol: May include investigators, veterinarians, breeders and owners	- Potential to examine a single issue in great detail	- Not quick
		- Diagnostic criteria set according to study protocol	- Potentially very expensive
		- May assess wider environmental exposures	- Recruitment not simple
			- Potential for retention bias in uncontrolled conditions
			- May only examine multiple exposures *OR* multiple outcomes
Prospective: Hypothesis generation	According to study protocol: Animals typically population-based but data maybe generated by investigators, veterinarians, breeders and owners	- May assess multiple exposures and outcomes including wider environmental exposures	- Not quick
		- Diagnostic criteria set according to study protocol	- Not cheap
		- Potential to describe health and lifestyle of current population	- Delay to results and lack of specific focus make funding difficult
		- Potential to assess the broad impact of lifestyle on disease	- Recruitment not simple
		- Potential to generate new hypotheses	- High susceptibility to retention bias
			- Potential for poor diagnostic accuracy if reliant on owner-reporting

### Retrospective methods

Retrospective cohort studies involve looking back at individuals after the events of interest have occurred (for example disease incidence, death or pregnancy) and the follow-up period has ended [21]. These studies can be undertaken on a large scale with relatively little lead time or up-front costs by using pre-existing databases such as those maintained by insurance companies and groups of secondary veterinary hospitals or primary clinics.

Insurance databases in particular are an extremely valuable resource and are discussed in detail by O’Neill *et al.*[22]. There is a long tradition of pet insurance in Sweden and Agria insure approximately 40% of Swedish dogs [23]. Their database provides a powerful measure of events in the Swedish pet canine population [24]. Such large electronic resources offer the chance to study incidence rates and survival time from diagnosis for specific diseases, such as mammary tumours [25]. However there is no requirement for private companies to make their data available. When using insurance databases, there is likely to be bias relating to the non-random socioeconomic status of owners who insure their pets and to specific insurance policy exclusions such as pre-existing conditions and age limits. In addition, in countries where dog insurance rates are low, the resource would be even less representative of the population as a whole.

Veterinary medical databases provide an alternative resource of information on the health of populations [22]. They have the advantage of that they can be linked to ancillary resources (such as radiographic archives and biological samples). However, the plethora of recording systems, and lack of agreement of diagnostic criteria for the definition of specific diseases, makes them cumbersome to use and extracting and extrapolating data is difficult. With modern textural mining tools there is scope to revisit this area [22] but the challenge of collating records from diverse recording systems remains. Further, when these databases rely solely on groups of specialist hospitals, there is the risk of referral bias as demonstrated by Bartlett *et al.*[26].

Risk factor studies using both insurance and veterinary medical databases are also limited by the type of data collected. In both cases, the data refer to phenotype of the dog but not their wider environment. Postcode (location) data have been used to assess the spatial distribution of atopic dermatitis [27] but the impact of the dogs’ lifestyles is not available from such records. For example Glickman *et al.*[28] were able to investigate a link between severity of periodontal disease in dogs and subsequent chronic azotemic kidney disease (kidney disease causing high levels of blood urea and creatinine) because both diseases were recorded in clinical records, but environmental risk factors like diet could not be considered. This is a major limitation of such databases; otherwise their data on multiple disease outcomes, covering large numbers of dogs from different breeds, would be unparalleled in terms of potential for use in investigations.

### Prospective methods

Prospective studies are set up before the outcome of interest occurs and allow investigators to pre-select study subjects and specifically determine which data they wish to collect [21].

### Prospective methods: time-limited

Limiting the time at risk has been used to minimise the costs of studies where pre-existing data are not available. This also helps reduce bias through loss to follow up. A wealth of investigations have utilised this methodology, such as those investigating the spread of Leishmaniasis and other vector borne diseases in dog cohorts. Studies investigating disease incidence [29–31], detection methods [32–36] and the impact of a culling regime [37] have all used this approach. Cohort methodology was necessary in each study but cost minimisation and swift reporting were facilitated by limiting the follow-up times to from one to three vector seasons.

### Prospective methods: single factor

If time is not constrained, then the focus or numbers of dogs in a study may be narrowed. Perhaps the best example of this comes from a study of dietary restriction using a small group of Labrador Retrievers (48 dogs) in an experimental setting. This controlled trial has yielded an array of findings on the effect of dietary restriction on mortality [38, 39], immune function [40], and developmental joint disease [41–49]. The time-span and depth of this trial (including blood sampling and radiography at regular intervals) made it prohibitively expensive to perform on a larger scale but data on specific aspects, such as the life-long progression of osteoarthritis, could only be collected by following a cohort longitudinally in this manner.

Dobson *et al.*[50] undertook a study with a similarly narrow focus but were able to recruit dogs from the normal pet population in the UK. Following 174 flat-coated retrievers for up to 10 years they investigated the impact of neoplasia on mortality in that breed. Costs were also minimised in this case by contacting recruited owners just once per year for a health update and asking them to proactively contact the investigators if their dog fell ill. The study demonstrated that over 40% of the dogs died as a result of neoplasia, reducing their lifespan by three years compared to those that died from other causes.

Recruiting a large enough cohort to give the required power for an investigation and retaining that cohort to minimise bias are both key to the success of population-based cohort studies. Thrusfield *et al.*[51] studied a cohort of bitches for up to five years in an attempt to assess the impact of neutering on urinary incontinence. The onus for recruiting and maintaining the cohort was placed on volunteering veterinary surgeons. Perhaps because of this responsibility, some difficulty was encountered recruiting veterinarians to participate; whilst 233 initially agreed, only 16 went on to return data (a 7% response rate). The authors made every effort to minimise bias through randomisation techniques but the potential impact of selection bias on the study should not be overlooked.

Each veterinarian was asked to recruit 40 female puppies from their practices. Should these bitches subsequently become incontinent then they were no longer followed, whilst, by design, the remaining (continent) cohort were to be followed for five years. The veterinarians received letters encouraging them to continue with the study at one and three years, and a request to contact the involved owners to check that their dogs were not incontinent after five years. The authors cite slow initial recruitment as the main reason why only 504 dogs from an original 809 were followed for the full five years. They do not directly address how many of the remaining 305 dogs were lost to follow-up (only 22 developed incontinence), but the potential impact of retention bias on their results cannot be ignored. Nevertheless, by focussing on a single phenotype and spreading the responsibility for dealing with recruited animals amongst a number of veterinarians, it was possible to follow enough dogs to determine that neutered bitches had a risk of urinary incontinence that was nearly eight-fold that of intact bitches.

### Prospective methods: hypothesis generation

Beyond studies that focus on one disease or one exposure, there has been a movement in canine epidemiology toward the broader studies undertaken in human medicine such as the example of ALSPAC mentioned above [9]. These studies do not necessarily aim to test a single hypothesis but rather gather data to identify new areas of investigation. In canine medicine, questionnaires have been developed that cover a wide range of potential exposures and disease outcomes and they are directed at breeders, owners and veterinarians. These studies have the disadvantage of relying on non-standardised data inputs where each animal is assessed by a different person with disparate (or no) training. However the studies are able to recruit more participants, and their subjects are more representative of dog lifestyle in the wider population than those followed under controlled conditions.

A 10-year cohort study of pedigree Boxers in the Netherlands recruited over 90% of the litters born in 14 months of 1994–5, initially comprising 2629 puppies. The study used diary-format records and face-to-face assessment with the breeders but moved on to six-monthly questionnaires with owners. Pre-weaning mortality [52, 53] and post-weaning mortality [54] were assessed and, due to the large numbers of dogs involved in the study, all with pedigree information, the investigators were able to make heritability estimates for phenotypes [55] and common diseases such as cryptorchidism (failure of one or both testes to descend to the scrotum), cranial cruciate disease (degeneration of the cranial cruciate ligament) and epilepsy [56] (a neurological disease characterised by the development of seizures) and hip dysplasia (a developmental malformation of the hip joints) [57].

Similarly a group in Norway followed a cohort of 700 dogs from four large breeds. Again they gave questionnaires to breeders and owners but they also involved the dogs’ veterinarians. To date they have published studies on the prevalence and risk factors of neonatal mortality [58], the effect of weight and growth rates on the development of hip dysplasia [59] and the incidence and risk factors associated with vomiting and diarrhoea [60].

Relatively newly created is the Dogslife Project [61], which is focussed on the owners of Kennel Club registered Labrador Retrievers in the UK [62]. It is limiting costs by utilising a website-based questionnaire and has recruited over 4,200 dogs in three and a half years. As a prospective study, it was possible to specifically tailor the questionnaire to address areas of interest. Data collection includes detail regarding phenotype and lifestyle which will be examined with reference to dog health. Like the studies in Norway and the Netherlands, the Dogslife Project is an attempt to develop a large-scale cohort of dogs with thoroughly documented history, similar to those cohorts found in human medicine.

## The future of canine cohort research

With the relative dearth of cohort studies in canines to date, there is scope to address new questions in the future. For example, the cohort of Dutch Boxer dogs discussed earlier were reported to have a pre-weaning mortality rate over 20% [52]. Such a loss is a clear welfare problem for dogs and more detailed studies of potential risk factors could have a great impact. Indrebø *et al.*[58], Nielen *et al.*[52] and van der Beek *et al.*[53] each address early mortality through cohort studies but their findings focus largely on factors from birth onward. Van der Beek *et al.*[53] included an analysis of inbreeding coefficients but found that genetic effects in general had less effect than environmental effects at puppy and litter level. Relatively short cohort studies including the lifestyle of the dam prior to birth may shed new light on risk factors associated with both still-births and early mortality, minimising distress in owners who are currently unable to prevent early losses.

Cohort studies such as those undertaken in human medicine could play a vital role if veterinarians are to be able to offer advice to owners on minimising the risks of developing disease and injury. Beyond death in very early life, morbidity and mortality in dogs in developed nations reflects the epidemiological shift in morbidity and mortality in human medicine from infectious diseases to non-communicable diseases. This shift is increasingly relevant in canine health as vaccination, antibiotics and better veterinary care ensure that more dogs in developed nations live to suffer from developmental diseases and diseases of aging. Bonnett *et al.*[63] demonstrated that whilst the highest mortality rate in dogs over six weeks of age in Sweden was trauma (typically car accidents), the next highest rate was due to tumours, followed by locomotor problems. Cohort studies of canine lifestyle have the power to investigate the risk factors associated with developing these non-communicable diseases, facilitated by the release of a draft canine genome sequence [64, 65] and the increasing access to high density genotyping and eventually low cost whole genome sequencing. Since the dog has a shorter lifespan than humans, associations between genetic variation and disease that are also relevant to human aging are likely to be revealed.

Human medicine is again ahead of the veterinary field with regard to incorporating biological data in cohort studies. UK Biobank that have recruited 500,000 people between 40–69 years of age. The investigative team collect blood, saliva and urine samples, phenotypic data and the agreement of all participants to have their health status followed. The collection of genetic information in particular adds a new element to the traditional cohort study, and with such a large cohort the potential power to detect risk factors involving genetic-environmental interactions is enormous. Projects on such a scale are currently financially prohibitive in dogs, but projects on a smaller scale such as Dogslife [61] have collected buccal swabs for DNA extraction from a subset of their cohort enabling comparisons of genotype with phenotype. Should such sampling be repeated throughout the lives of the dogs, investigations of genetic and epigenetic changes throughout that lifetime and comparison with concurrent phenotypic data would potentially permit investigators to seek environmental factors associated with genetic variation, disease and aging. The merging of lifestyle and whole genome data should increasingly reveal associations between genotype and environment in the dog and ultimately in humans as well.

## Conclusions

Cohort studies have already yielded results in the field of canine health. With the advent of large databases and internet technology the costs of such studies are being reduced to the point whereby large-scale studies are possible in canine populations. The potential to identify risk factors and inform an evidenced-based medicine approach to preventative health measures in dogs mean that cohort studies can have a great impact on dog health and welfare. Given how long it takes to achieve results from prospective studies, the time to start is now.

## Abbreviations

ALSPAC: Avon longitudinal study of parents and children
HRT: Hormone replacement therapy.
